# Musculoskeletal disorders among orthopedic pediatric surgeons: an overlooked entity

**DOI:** 10.1007/s11832-016-0767-z

**Published:** 2016-08-18

**Authors:** Mohammad M. Alzahrani, Saad M. Alqahtani, Michael Tanzer, Reggie C. Hamdy

**Affiliations:** 1 Division of Orthopaedic Surgery, Shriners Hospital for Children, Montreal Children Hospital, McGill University, 2000 Drummond, Apt.1203, Montreal, QC H3G 2X1 Canada; 2 Division of Orthopaedic Surgery, McGill University, Montreal, QC Canada; 3 Department of Orthopaedic Surgery, University of Dammam, Dammam, Saudi Arabia

**Keywords:** Musculoskeletal, Pediatric, Orthopedic, Disorder

## Abstract

**Introduction:**

Forceful and repetitive maneuvers constitute the majority of pediatric orthopedic surgical tasks, thus subjecting surgeons to the risk of musculoskeletal (MSK) injuries during their years in practice. The aim of this study was to assess the prevalence, characteristics and impact of MSK disorders among pediatric orthopedic surgeons.

**Methods:**

A modified version of the physical discomfort survey was sent to surgeons who were members of the Pediatric Orthopedic Society of North America (POSNA) via e-mail. The collected data were analyzed using descriptive statistics, one-way analysis of variance, and Fisher’s exact test. *p* values of <0.05 were considered statistically significant.

**Results:**

Of the 402 respondents, 67 % reported that they had sustained a work-related MSK injury, of which the most common diagnoses were low back pain (28.6 %) and lateral elbow epicondylitis (15.4 %). Among those which reported an injury, 26 % required surgical treatment and 31 % needed time off work as a direct result of their injury. The number of work-related injuries incurred by a surgeon increased significantly with increasing age (*p* < 0.001), working in a non-academic institute (*p* < 0.05), working in more than one institute (*p* < 0.05), and being in active practice for >21 years (*p* < 0.05). The need to undergo treatment or take time off due to the injury was associated with increased number of injuries (*p* < 0.001). In addition, surgeons were more likely to require time off work when they were >56 years of age (*p* < 0.001), had been in practice for >21 years (*p* < 0.001), required surgical management of their disorder (*p* < 0.001), and had experienced an exacerbation of a previous disorder (*p* < 0.001).

**Discussion and conclusion:**

This study is the first of its kind to assess MSK injuries sustained by pediatric orthopedic surgeons. The high incidence of these disorders may place a financial and psychological burden on these surgeons and thus the healthcare system. These results should shed a light on awareness and the need for further studies to prevent and help decrease the incidence of these disorders not only in orthopedic surgeons but also in the surgical population in general.

## Introduction

The majority of studies in the surgical literature have focused on improving the quality of care of the patients and implementing guidelines to provide a safe and adequate patient environment. Although this is of uttermost importance for both the patient and the healthcare system, the safety and well-being of the surgeon are also important, and this aspect of healthcare has only recently gained attention. The surgeon faces a wide range of occupational hazards in his/her work environment, specifically in the operating room, including exposure to potential chemical, radiation, and physical hazards as well as emotional stress [1, 2]. Psychologically, surgeons in general suffer from decreased sleep due to long working hours, especially during the nighttime, which recent studies have shown have an adverse effect on both the physical and mental health of these physicians [3, 4]. The orthopedic surgeon in particular works in close proximity to radiation from intraoperative fluoroscopy, and a 25-fold increase in thyroid cancer has been reported in spine surgeons [5].

Recent attention has been directed towards musculoskeletal (MSK) hazards that orthopedic surgeons are susceptible to during their practice [6]. The nature of the operative procedures in orthopedic surgery demands a specifically high level of strength and stamina from practicing surgeons [7]. Studies have shown an alarmingly high incidence of MSK disorders in surgeons in various subspecialties of orthopedic surgery, which has been attributed to the high incidence of repetitive movements and prolonged periods of sustaining ergonomically abnormal positions [7–10]. Although limited in number, guidelines are available to improve this working environment, but global implementation of these recommendations has been prevented due to the many hurdles that have been encountered [11–13].

Pediatric orthopedic surgery is one of the subspecialties of orthopedic surgery that demands an increased workload on the surgeon, specifically during spine and hip procedures. Therefore, we undertook a study to determine the prevalence of MSK injuries in pediatric orthopedic surgeons, to identify specific risk factors for these injuries, and to determine their impact on the practice of the surgeon.

## Methods

A modified version of the physical discomfort survey was sent to all surgeons who were members of the Pediatric Orthopedic Society of North America (POSNA) via e-mail after ethics committee approval. An initial e-mail was sent in December 2014, a reminder was sent February 2015, and the survey was closed by the end of March 2015.

The survey included questions on the surgeon’s general demographics (e.g., age, gender, hand-dominance, type of practice, number of years in practice, annual caseload) and on work-related MSK injuries. The questions on the latter were divided into sections according to anatomical region (neck, shoulder, elbow/forearm, wrist/hand, hip, knee, foot and ankle, low back), and for each of these regions the participants in the survey were asked about the treatment required and the amount of time off work required due to the injury, if any. Prior to sending out the survey to the surgeons, we conducted a pilot study involving ten volunteers was to assess the length of time required to complete the survey, the comprehensiveness of the questions, and the ease of navigation through the survey.

The collected data were analyzed using descriptive statistics, one-way analysis of variance and Fisher’s exact test. *p* values of <0.05 were considered to be statistically significant.

## Results

**Fig. 1:**
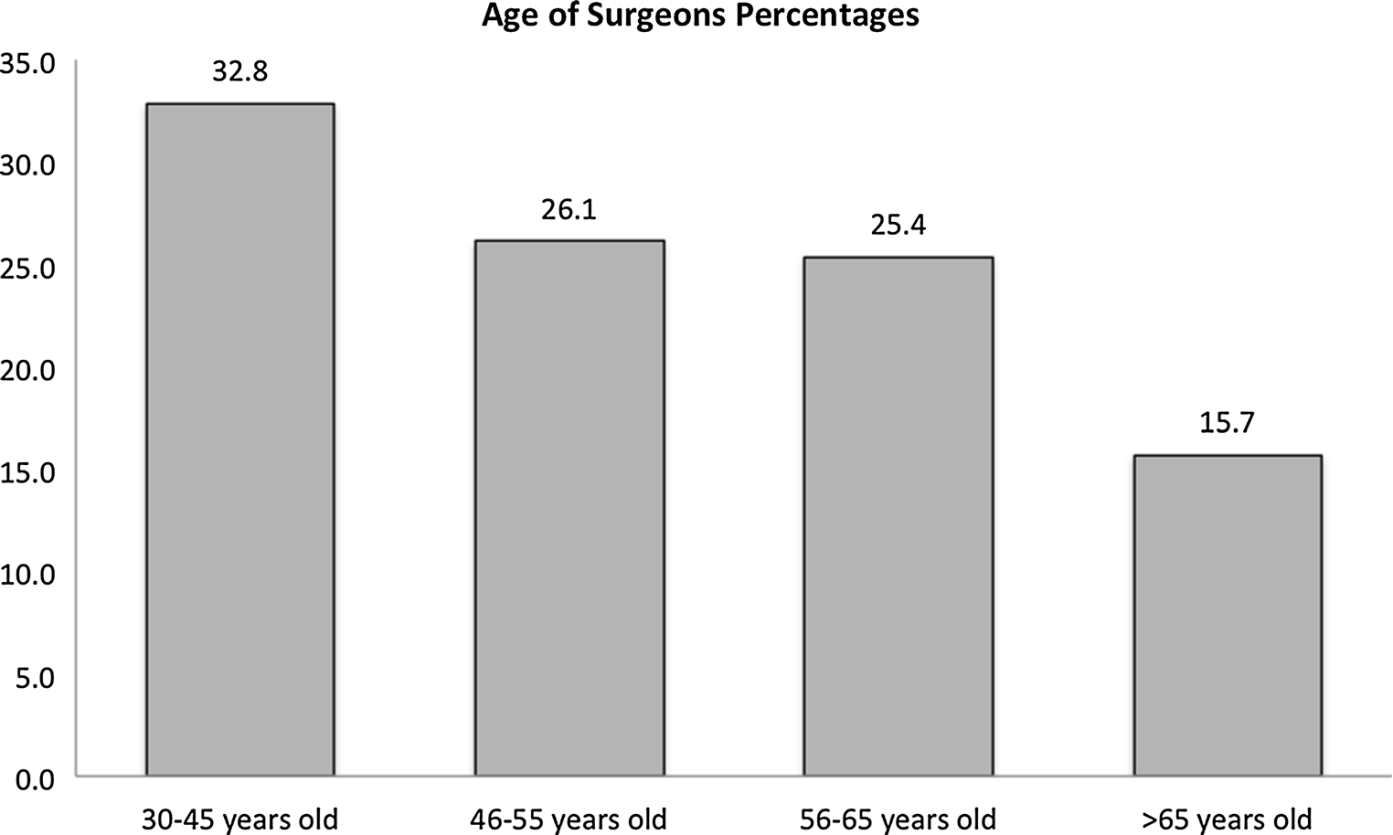
Age distribution of participating (responding) pediatric orthopedic surgeons

**Fig. 2:**
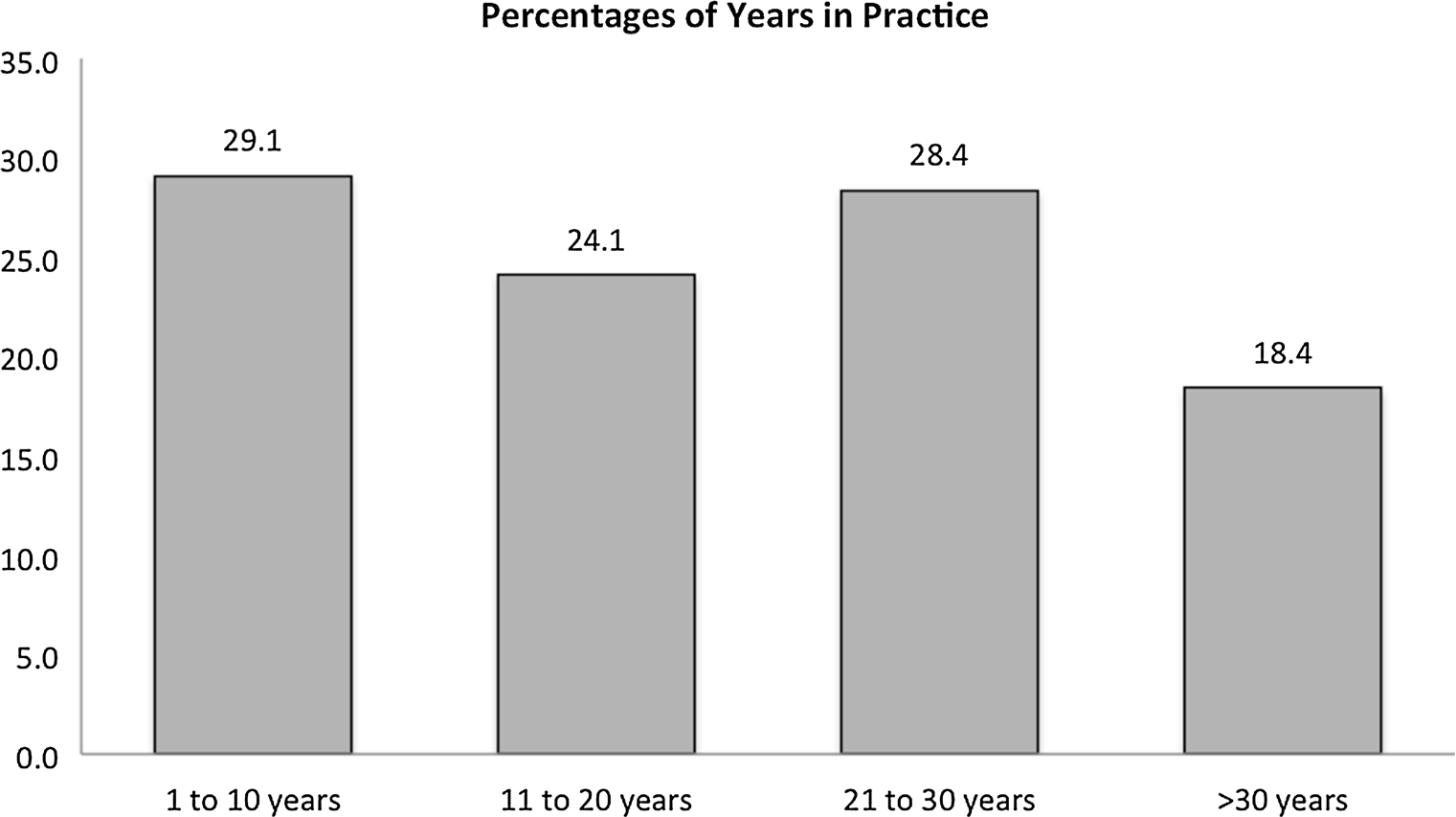
Distribution of years in practice of participating pediatric orthopedic surgeons

A total of 402 surgeons completed the survey during the period of data collection, with a respondent rate of 31 %. Of the respondents, 76 % were male, >84 % were ≤65 years of age, and 82 % were in practice for ≤30 years (Figs. 1, 2; Table 1). The majority (73 %) of the respondents were working in an academic institute, and 8 % worked in more than one institute. Overall, 67 % of the respondents reported that they had sustained a work-related MSK disorder at some point during their career, among whom 26 % required surgical treatment and 31 % needed time off work due to their injury (Tables 2, 3). In addition, 22 % of the surgeons reported that they had an exacerbation of an MSK disorder that was previously present.

**Table 1:** Demographics of surveyed pediatric orthopedic surgeons

Variables	Percentage
Total respondents	100
Sex	
Male	76
Female	24
Hand dominance	
Right	90
Left	10

**Table 2:** Percentage of surveyed pediatric orthopedic surgeons with injuries and time off work requirement according to demographics and type of practice

Variables	Percentage of respondents with injuries	Percentage of injured respondents requiring time off work
Age (years)		
≤45	62	15
46–55	74	28
56–65	72	47
>65	59	38
Sex		
Male	66	29
Female	71	36
Hand dominance		
Right	68	31
Left	63	27
Type of practice		
Academic	65	30
Community	74	44
Private	77	13
Other	71	42
Number of institutes		
1	67	29
>1	69	46

**Table 3:** Percentage of surveyed pediatric orthopedic surgeons with injuries and time off work requirement according to annual case load and number of years in practice

Variables	Percentage of respondents with injuries	Percentage of respondents with injuries requiring time off work (%)
Annual caseload		
≤100	48	35
101–200	63	27
201–300	71	29
301–400	72	30
401–500	75	27
>500	80	45
Years in practice		
≤10	62	11
11–20	68	21
21–30	76	51
>30	61	38

**Fig. 3:**
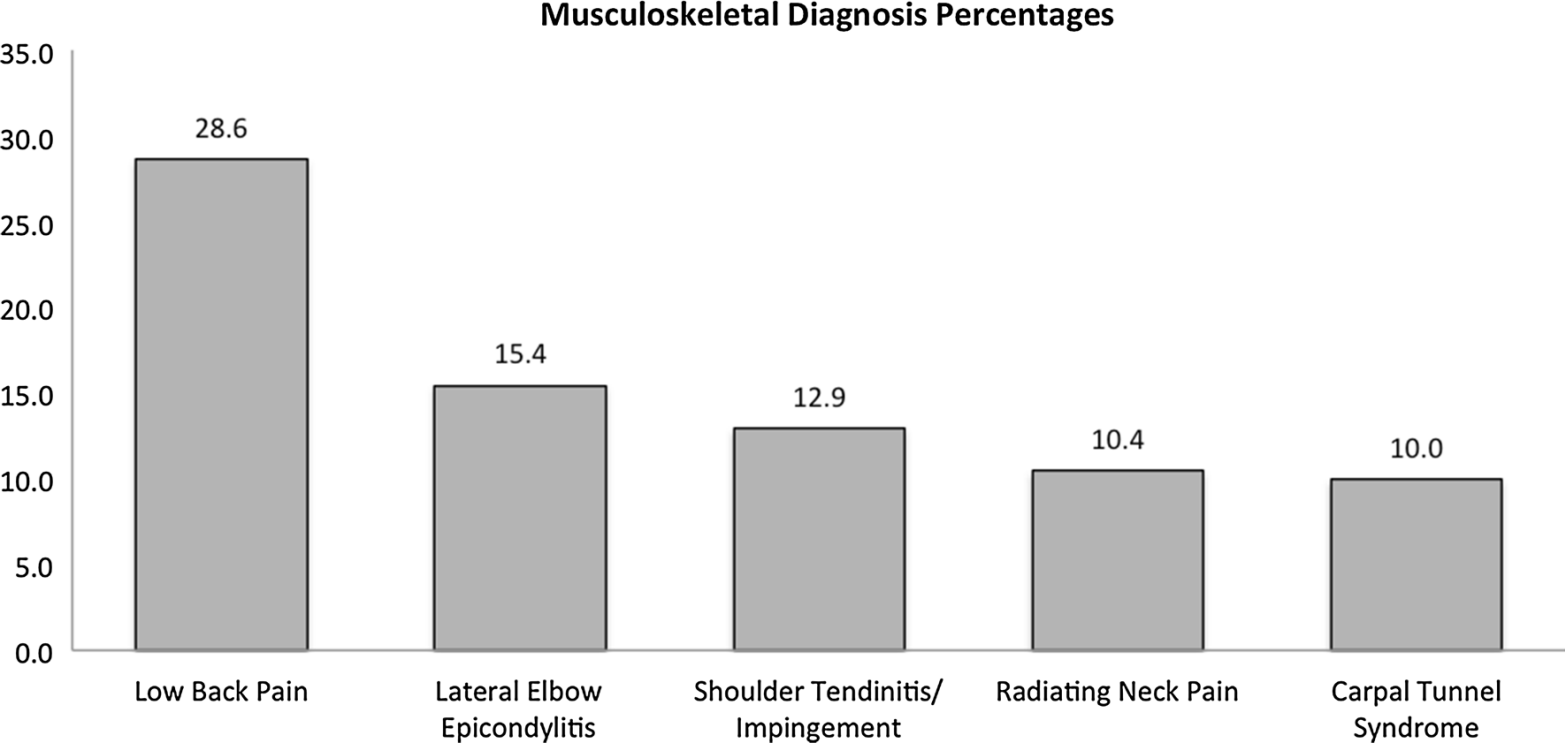
Prevalence of the most common musculoskeletal disorders among participating pediatric orthopedic surgeons

In terms of the anatomical regions involved in the MSK discorder, the lower back and upper extremity were the two most commonly injured areas, with 29 % of respondents reporting low back pain as a MSK work-related injury and 15 % developing lateral epicondylitis of the elbow as a result of their work (Fig. 3; Table 4). Non-MSK disorders were also reported by the surgeons, with the most common being varicose veins (8 %) and inguinal hernia (5 %). When we examined each anatomical region separately, we found that the lower back was the most common region reported (44 % of respondents reported a lower back disorder, which includes low back pain, spondylosis, degenerative disc disease, disc herniation, and stenosis) and that the foot and ankle region was the most common region requiring treatment in general (75 % of injured respondents). The knee and lower leg region was the most likely region to require surgical treatment (34 % of injured respondents) and the most common with respect to requiring time off work (32 % of injured respondents) (Table 4).

**Table 4:** Percentage of surveyed pediatric orthopedic surgeons with diagnosed injuries categorized by anatomical region and percentage of general treatment, surgical treatment and time off work requirement due to their musculoskeletal injuries

Region	Percentage of respondents with injuries	Percentage of injured respondents requiring treatment	Percentage of injured respondents requiring surgical treatment	Percentage of treated respondents requiring time off work
Neck	24	52	10	16
Shoulder	23	36	14	18
Elbow	18	59	7	6
Forearm, wrist and hand	34	47	23	24
Hip and thigh	7	46	19	19
Knee and lower leg	11	61	34	32
Foot and ankle	9	75	8	8
Lower back	44	57	12	21

The number of injuries showed a significant increase with increasing age (*p* < 0.001), working in a non-academic institute (*p* < 0.05), working in more than one institute (*p* < 0.05), and being in clinical practice for >20 years (*p* < 0.05). The likelihood that a surgeon would require treatment or take time off due to an injury increased with the number of injuries sustained (*p* < 0.001). We also found that surgeons who were aged >55 years (*p* < 0.001), had been in clinical practice for >20 years (*p* < 0.001), required surgical management of their disorder (*p* < 0.001), and experienced an exacerbation of a previous disorder (*p* < 0.001) were more likely to require time off work (Table 2, 3). Exacerbation of a previous MSK injury was more common in female surgeons (*p* < 0.05), surgeons working in a community setting (*p* < 0.001), surgeons working in more than one institute (*p* < 0.001), and surgeons with an increased number of reported injuries (*p* < 0.001).

## Discussion

The aim of this study was to investigate work-related injuries in pediatric orthopedic surgeons and the effect(s) of such injuries on the affected surgeon’s practice. Overall, a high percentage of surgeons who completed the survey (67 % of respondents) reported a work-related MSK injury during their practice, with 26 % of these surgeons requiring surgical treatment for their injury and >31 % requiring time off work as a consequence of the injury. These alarmingly high numbers indicate that pediatric orthopedic surgeons are in a high-risk profession and are exposed to hazards associated with current operating room environments and surgical equipment.

The prevalence of injuries found in our study is similar to that reported in previously published surveys of orthopedic surgeons. Alqahtani et al. reported that a work-related injury occurred in 66 % of arthroplasty surgeons and in 67 % of orthopedic trauma surgeons [9, 10]. Davis et al. found a lower prevalence of injuries, 44 %, in 140 orthopedic surgeons of different surgical specialties [7]. In all three studies, the most commonly reported injured anatomical areas were the lower back, upper extremity, and neck. This was also the case in a study by Auerbach et al. of 561 spine surgeons, among whom the lower back and neck were most commonly injured areas [8]. Our study produced similar results, as the most common areas injured in our cohort were the lower back and elbow. The high prevalence of injuries in these regions can be associated with the tendency to sustain constant positions of back flexion while performing the operative procedure, as well as the need to carry out repetitive manual tasks with the wrist extensors, thereby applying increased strain on these regions. Compared to the general population, pediatric orthopedic surgeons have a higher incidence of low back pain (29 vs. 6–10 %) and lateral epicondylitis of the elbow (15 vs. 0.4–1.3 %) [14–17].

Interestingly, we found that increasing age (>55 years), increasing number of years in practice (>20 years), and requirement for surgical management of the injury were associated with an increased number of reported injuries and the need to take time off work due to the sustained injuries. In their study of orthopedic trauma surgeons, Alqahtani et al. also found that increasing age and being in practice for >10 years were associated with an increased number of injuries diagnosed [9]. These authors also showed that working in a private setting and working in more than one institute—but not age nor years in practice—were more likely to require time off work due to injuries [9]. This was not the case in Alqahtani et al.’s study of arthroplasty surgeons; these authors found that increasing age (>55 years old) and increasing years of clinical practice (>20 years) were associated with an increasing requirement to take time off work due to injuries, but not with the number of reported injuries [10].

In our study, female gender, working in more than one institute, community practice, and increased number of injuries were risk factors for exacerbation of a previous injury. In previous studies, exacerbation of a previous injury was only associated with an increased number of disorders in the arthroplasty surgeon study [10]. Surprisingly, we found no effect of the annual case load on any of our outcomes (number of disorders, requiring time-off work and exacerbation of a previous injury), similar to the results on trauma surgeons reported by Alqahtani et al. [9]. On the other hand, in a study on arthroplasty surgeons, Alqahtani et al. did find that performing >100 total hip arthroplasties per year was a risk factor for requiring time off work due to an injury [10].

The likely culprits of the high rate of work-related injuries found in this study are most likely the operating room environment and the surgical equipment. The operating room environment has been shown in previously published studies to have a crucial effect on the physical and mental health of the surgeon [7, 8]. Therefore, this aspect of surgeon safety should play a more important role in the healthcare system. Improved ergonomics in the operating room and surgeon educational programs should be more implemented, including recommendations for a distribution of work-load with assistants in the operating theater and the taking of short breaks during long operative procedures [8, 18, 19]. A number of physical modalities can be implemented, such as the use of ergonomic body supports, compressive stockings to reduce the effect of dependent edema during long periods of standing, and the use of power tools instead of manual tools when applicable [2, 11].

There are several limitations to our study. First, as in all surveys, the reliability of self-reported injuries in this survey could not be established clearly and the effect of recall bias cannot be ignored. Also, although these injuries were attributed by the respondents to the work place, other contributing factors may have an effect, including physical activities outside the hospital, which may have been the culprit behind these reported injuries. In addition, we did not examine the effect of other aspects that may have made a significant contribution to these injuries, including time and case load of a clinic or operating room day.

## Conclusion

This study is the first of its kind to assess MSK injuries sustained by pediatric orthopedic surgeons. We found a high incidence of these disorders in our population of orthopedic surgeons, which may play a financial and psychological burden on these surgeons and thus the healthcare system. This study should shed a light on awareness and the need for further studies aimed at preventing and decreasing the incidence of these disorders not only in orthopedic surgeons but also in the surgical population in general.
